# Comparative transcriptome analysis and candidate gene mining for fire blight of Pear resistance in Korla fragrant Pear (*Pyrus sinkiangensis* Yü)

**DOI:** 10.1038/s41598-025-00009-2

**Published:** 2025-04-29

**Authors:** Yue Li, Yuanrong Ye, Wei Huan, Juan Ji, Jieyun Ma, Qiang Sheng, Jianfeng Lei

**Affiliations:** 1 College of Life Sciences, Xinjiang Agricultural University, Nongda East Road, Urumqi, 830052 China; 2 Academy of Agricultural Sciences of Bayinguoleng Mongolian Autonomous Prefecture, Yinxia Road, Korla, 84100 China; 3 College of Agriculture, Xinjiang Agricultural University, Nongda East Road, Urumqi, 830052 China

**Keywords:** Korla fragrant Pear, Fire blight, RNA-seq, WGCNA, Candidate genes, Gene expression profiling, RNA sequencing

## Abstract

Fire blight of pear is caused by Erwinia amylovora, which can cause devastating damage to pear trees and other pome fruit trees worldwide. Fire blight has resulted in a reduction in the production of Korla fragrant pears in Xinjiang, China, as well as a decrease in their quality and taste, causing severe economic losses. To this end, a comparative transcriptome analysis of common Korla fragrant pear (KFP, susceptible) and a bud mutation line (1910, resistant) at three inoculation periods was conducted. Clustering and principal component analysis (PCA) of the RNA-seq data revealed that the differences between lines were greater than those within lines. A total of 7271 DEGs were identified in the bud mutation line, while 11,937 DEGs were identified in KFP. Between KFP and the resistant material bud mutation line, 11,937 DEGs were identified, which were significantly enriched in the pathways of photosynthesis, jasmonic acid metabolic process, flavonoid biosynthesis, and starch and sucrose metabolism. A total of 8 clusters were identified for all (17,354) DEGs via k-means, and KEGG pathway annotations were performed for each individual cluster. In addition, the 1027 differentially expressed transcription factors (TFs) were clustered into five clusters, and the TFs with the largest fold change in each cluster were identified. A gene coexpression network was further constructed through weighted correlation network analysis (WGCNA), and 15 key genes that determine the fire blight resistance of Korla fragrant pear were identified. These research results provide a theoretical basis for a deeper understanding of the molecular mechanism of Korla fragrant pear resistance to fire blight and provide new genetic resources for the study of Korla fragrant pear resistance to fire blight.

**Supplementary Information:**

The online version contains supplementary material available at 10.1038/s41598-025-00009-2.

## Introduction

The origin of Korla fragrant pear is Xinjiang. It is a perennial fruit tree of the Xinjiang pear species (*Pyrus sinkiangensis* Yü) of the genus Pyrus in the Rosaceae family. Korla fragrant pear not only is refreshing, crisp, juicy, sweet, and durable during storage but is also rich in nutrients and has medicinal functions such as relieving cough, moistening the lungs, and reducing inflammation^1^. Therefore, it is loved by consumers and is a staple of the forestry and fruit industry in Xinjiang. Fire blight of pear is a devastating bacterial disease of Rosaceae fruit trees caused by *Erwinia amylovora*^2^. Since 1780, fire blight has been detected in more than 60 countries around the world, including Europe, North America, North Africa, the Middle East, Oceania and Asia^3^. In Xinjiang, China, pear fire blight has mainly broken out in Korla city and Yuli County, harming fruit trees such as fragrant pear and apple, as well as landscape trees such as crabapple and hawthorn^4^. Pathogens associated with amylopectin disease can spread over long distances. The initial infection can invade the host through flowers, natural openings and wounds^5^. Within the pear tree, it can migrate systemically through the intercellular spaces of the cortical parenchyma and can also spread through the xylem vessels. It quickly infects other healthy parts of the plant, causing flowers, leaves and young fruits to wither and turn black but not fall off^6^. After the initial infection, pear fire blight continues to occur throughout the growing period. The fungal pus on diseased pear trees becomes a secondary source of infection, quickly spreading to the entire orchard through insects, birds, wind and rain, pruning tools, etc. In autumn, branches infected with the pathogen will form ulcers. The pathogen overwinters in ulcers and infects pear trees again the following year^6^.

With the development of sequencing technology, the transcriptome has been widely used in the study of plant disease resistance^7–12^. Comparative RNA-seq analysis of the Corynespora leaf spot of the two sesame varieties revealed that 12 h postinoculation (hpi) was the key time point leading to the differences in resistance between the two lines^7^. Through RNA-seq analysis, it was found that the expression of the *HLP1* gene in rice was significantly upregulated in response to rice blast infection, and *OsHLP1*-overexpressing plants presented significantly increased resistance to rice blast^8^. Through RNA-seq and the identification of metabolite components of eggplants highly resistant and highly susceptible to Verticillium wilt, a total of 4017 differentially expressed genes (DEGs) were identified, and coexpression analysis revealed that 13 transcription factors (TFs) were key genes involved in the eggplant defense response^9^. Through RNA-seq of the highly susceptible cotton variety Junmian 1 and the highly resistant cotton variety Liaomian 38, it was found that most DEGs were annotated to resistance-related pathways^10^. RNA-seq analysis revealed that the early interaction between Chinese cabbage and clubroot caused significant changes in the expression of some defense genes, such as NBS-LRRs. Peroxidase (POD), salicylic acid (SA) and jasmonic acid (JA) are important signaling molecules for the resistance of Chinese cabbage to clubroot^11^. Comparative RNA-seq analysis of pepper infected with anthracnose revealed that plant hormone signaling pathways and the synthesis and metabolism of phenylpropanoids were activated. In addition, the WRKY and NAC transcription factors may play positive regulatory roles in the defense response against anthracnose^12^.

Fire blight is severe because of the single planting pattern, farming methods and agronomic measures used for Korla fragrant pear. The breeding of disease-resistant varieties is an important method for preventing and controlling pear fire blight^13^. The breeding speed of disease-resistant varieties can be accelerated by combining traditional breeding and molecular marker-assisted breeding^14^. Therefore, the most economical and effective way to solve the current problem of Korla fragrant pear is to explore the fire blight resistance genes of Korla fragrant pear, study the regulatory network of defense response genes, analyze the molecular mechanism, and cultivate disease-resistant lines and varieties. In this study, transcriptome sequencing was performed on common Korla fragrant pear (KFP, susceptible) and a bud mutation line (1910, resistant) inoculated with fire blight at different stages. Through differential expression analysis, cluster analysis, enrichment analysis, expression pattern analysis of hormone biosynthesis and signal transduction genes, WGCNA and qRT‒PCR, the key genes that induce Korla fragrant pear resistance to fire blight and the regulatory pathways related to defense were further characterized, revealing the mechanism of action of Korla fragrant pear resistance to fire blight. These results are highly important for the research and genetic engineering of Korla fragrant pear resistance to fire blight and provide theoretical support for the effective prevention and control of fire blight.

## Materials and methods

### Plant materials

This study used fragrant pear leaf samples from a fragrant pear orchard in Korla, Xinjiang (86°7′32″E, 41°44′25″N). KFP is a common fragrant pear, and 1910 was a bud mutation resource for fragrant pear. The test strains (*Erwinia amylovora* E. a001) were kindly donated by Professor Ming Luo from the Agricultural Microbiology and Biotechnology Laboratory of Xinjiang Agricultural University. Five healthy and uniform-growing fragrant pear trees and five fragrant pear bud mutation 1910 trees were selected at the experimental base of the Bayingol Academy of Agricultural Sciences in Korla city, Xinjiang, without taking any measures to prevent and control fire blight. The *Erwinia amylovora* (E. a001) strain was streaked onto NA + 5% sucrose media. A single colony was picked and inoculated into NB medium. Another bottle without bacteria was used as a control and placed in a shaker at 28 °C and 180 r·min^− 1^ for 24 h until the OD600 reached 0.6–0.8. The mixture was diluted with sterile water and shaken vigorously to obtain a pathogen suspension. A hemocytometer was used to count the spores, and the concentration was adjusted to 1 × 10^8^ CFU·mL^− 1^ under an optical microscope. One-year-old branches with consistent growth were selected and inoculated with an E.a001 suspension via the needle puncture method. A sterile toothpick was used to prick a wound approximately 0.5 cm approximately 3 cm away from the top of the young branch of the fragrant pear. A pipette was used to draw the bacterial suspension, which was then dropped into the wound. A circle of sterile cotton strips soaked in sterile water 1 cm above the inoculation point was removed, the inoculation site was wrapped together with the absorbent cotton strips with plastic wrap, the strips were placed in a bag to keep them moist for 24 h, and then the plastic film and absorbent cotton above the inoculation site were removed. Ten branches of each common fragrant pear (KFP) and fragrant pear bud mutation resource line (1910) were inoculated, and sterile water was used as a control. After inoculation, the branches were observed for disease on a regular basis every day, and the distance and time of lesion expansion on the branches of the diseased pear plants were measured and recorded. When inoculated, the ambient temperature should be 25–30 °C, which is the optimal temperature for the growth of E.a001. The appropriate ventilation conditions and sufficient sunlight on the branches and leaves of the inoculated branches were maintained to ensure the best inoculation effect and repeatability. Pear leaf samples were collected at 0 h, 24 h, and 72 h after inoculation, with three biological replicates per sample, rinsed, dried, wrapped in tin foil, quickly frozen in liquid nitrogen, and shipped on dry ice.

### RNA-seq library construction and sequencing

RNA was extracted via a polysaccharide and polyphenol plant total RNA extraction kit (each sample was approximately 100 mg, and each sample contained three biological replicates), and the extraction process was carried out according to the instructions. After RNA extraction, a Nanodrop was used to determine the RNA purity (OD260/280) and concentration and to determine whether the nucleic acid absorption peak was normal. The integrity of the RNA was accurately detected with an Agilent 2100 instrument. The detection indicators included the following: the RIN value, 28 S/18S ratio, whether the chromatogram baseline was increased, and the 5 S peak. After the samples passed the test, they were transported on dry ice to Xinjiang Aidesen Biological Co., Ltd. (Urumqi, China) for sequencing. Magnetic beads with oligo(dT) are used to enrich mRNAs by binding to the poly(A) tail of the mRNA through A-T complementary pairing. Then, fragmentation buffer was added to break the mRNA into short fragments. Random hexamers were used to synthesize first-strand cDNA using mRNA as a template. Then, buffer, dNTPs and DNA polymerase I were added to synthesize second-strand cDNA. The double-stranded cDNA was then purified via AMPure XP beads. The purified double-stranded cDNA was then end-repaired, A-tailed and connected to sequencing adapters, the fragment size was selected via AMPure XP beads, and finally, PCR enrichment was performed to obtain the final cDNA library. The library was detected via Agilent 2100 and qPCR methods. The libraries were sequenced on an Illumina HiSeq 2500 platform to generate 150 bp paired-end reads. Illumina HiSeq 2500 sequencing technology involves sequencing by synthesis (SBS), in which a fluorescently labeled reversible terminator is imaged as each dNTP is added and then cleaved to allow incorporation of the next base. Since all 4 reversible terminator-bound dNTPs are present during each sequencing cycle, natural competition minimizes incorporation bias. The method virtually eliminates errors and missed calls associated with strings of repeated nucleotides (homopolymers).

### RNA-seq analysis

After the original sequence was obtained, Fastp software (version 0.23.4) was used to remove the adapter sequence, filter out low-quality and N sequences with a ratio greater than 5%, and obtain clean reads that could be used for subsequent analysis^15^. Clean reads were aligned to the reference genome (https://www.rosaceae.org/species/pyrus_bretschneideri/genome_v1.1) via HISAT2 (version 2.2.1). R language PCAtools was used to decompose the expression data (FPKM) of all genes into n principal components to describe the characteristics of the original dataset^16^. PC1 represents the most obvious feature that can be described in the multidimensional data matrix, and PC2 represents the most significant feature that can be described in the data matrix except PC1. ggplot2 was used to visualize the PCA results.

### Differential expression analysis

We use featureCounts to compare and quantify the results. The unstandardized read count data were used as input data to calculate the P value and fold change value via DESeq2 software (Version 1.46.0), and the Benjamini Hochberg algorithm was used to correct the P value to obtain the FDR value. Generally, a |log_2_fold change| greater than 1 indicates that the expression of the gene has changed by at least one-fold, which is considered to be biologically significant. Setting the FDR standard to less than 0.01 ensures that fewer than 1% of the DEGs screened are caused by random differences. FDR < 0.01 and |log_2_fold change|>1 were used as the criteria for screening DEGs^17^. Enrichment analyses of the DEGs via Gene Ontology (GO) (https://www.geneontology.org/) and Kyoto Encyclopedia of Genes and Genomes (KEGG) (https://www.genome.jp/kegg/) analyses were conducted via the clusterProfiler software (version 4.14.4) package in R^18^. GO and KEGG analyses were performed via ClusterProfiler (version 4.14.4) with the hypergeometric test method, and a q value < 0.01 was selected to identify significant biological processes, cellular components, molecular functions, and KEGG pathways.

### WGCNA

The WGCNA package (version 1.73) in R was used to perform coexpression analysis on the DEG expression profile via the dynamic branch cutting method^19^. The weighting coefficient β should satisfy the correlation coefficient close to 0.8. In this study, when the power significance is greater than 0.8, the minimum value of β is 13, and β = 13 was selected as the weighting coefficient. The network was constructed via blockwise modules to obtain the gene coexpression modules (minModuleSize = 30 and Merge Cut Height = 0.25). The correlation coefficient and P value between the module characteristic vector ME (Module Eigengene) and Verticillium dahliae cultured at different temperatures were calculated. The coexpression network was visualized via Cytoscape software^20^.

### qRT‒PCR

Total RNA was extracted via a FastPure Plant Total RNA Isolation Kit (Novagen Biotech Co., Ltd., Nanjing). cDNA was synthesized via the use of PrimeScript Reverse Transcriptase (TaKaRa, Dalian). Primer Premier 5.0 software was used to design the qRT‒PCR primers (Table S1), with the pear *Actin* gene used as the internal reference. qRT‒PCR was performed using ChamQ Universal SYBR qPCR Master Mix (Novagen Biotech Co., Ltd., Nanjing). The reaction program was as follows: 95 °C predenaturation for 30 s, 95 °C denaturation for 10 s, 60 °C annealing for 30 s, and 72 °C extension for 20 s for 40 cycles. Each PCR was repeated 3 times. The quantitative data were analyzed via the 2^–∆∆CT^ method^21^.

### Source of plants statement

The chieh-qua varieties used in the study complied with relevant institutional, national, and international guidelines and legislation. The study also complies with the IUCN Policy Statement on Research Involving Species at Risk of Extinction and the Convention on the Trade in Endangered Species of Wild Fauna and Flora for the collection of seed samples used in the study. All samples were collected with permission from the Bayingol Academy of Agricultural Sciences Institute in Korla city, Xinjiang.

## Results

### RNA-seq data analysis

**Fig. 1:**
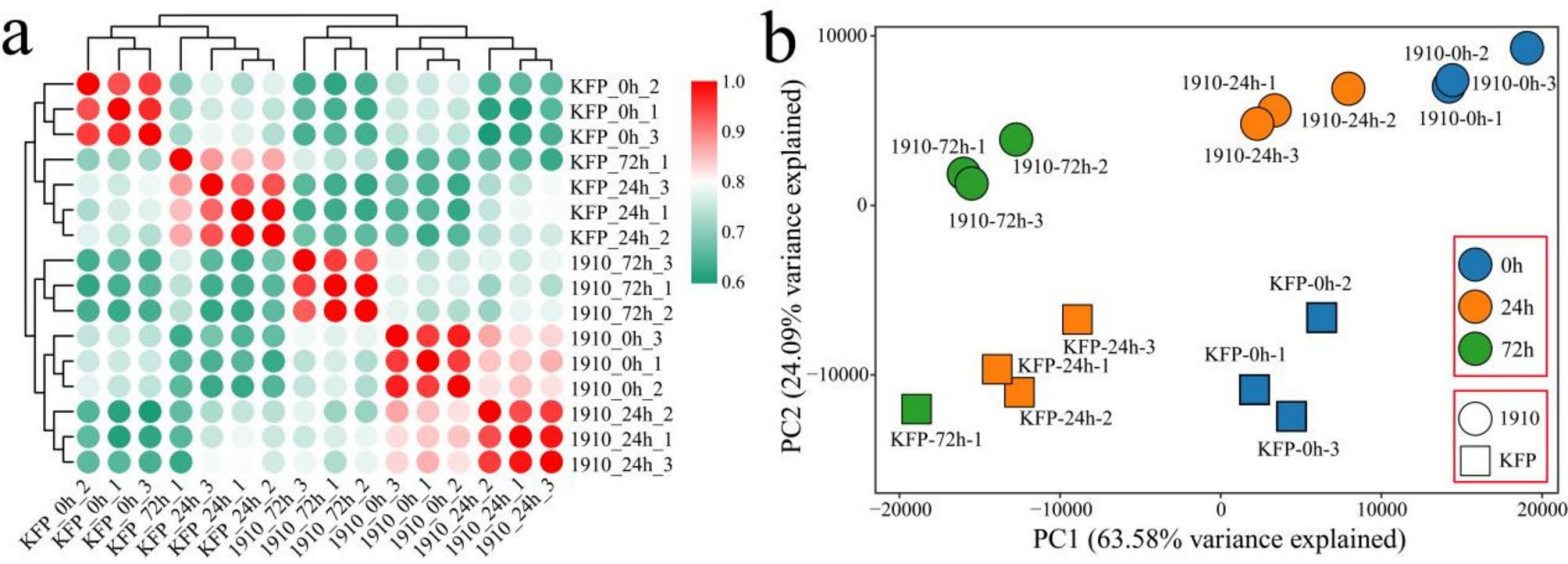
Correlation and PCA of 16 RNA-seq samples of Korla fragrant pear inoculated with fire blight. (**a**) Correlation analysis of samples, (**b**) PCA of samples.

A total of 110.12 Gb of clean data were obtained from 16 samples of common Korla fragrant pear (KFP, susceptible) and a bud mutation line (1910, resistant) inoculated with E. a001 (sequencing of 2 samples failed). The amount of clean data for each sample exceeded 5.84 Gb, and the Q30 base percentage was greater than 90.44% (Table S2). First, the correlation between three biological replicates of the same sample was calculated, and the correlation coefficients were all greater than 0.93 (Fig. 1a). The results of cluster analysis and principal component analysis (PCA) revealed that samples with the same biological replicates were clustered together, and KFP and 1910 at the same inoculation period could be distinguished, indicating that the material selection was reliable and that the transcriptome sequencing data were reliable and reproducible (Fig. 1a-b). The samples of the disease-resistant KFP line and the disease-resistant line 1910 inoculated with fire blight at different times were clustered together, indicating that the differences between lines were greater than the differences within the lines.

### Analysis of differential expression within lines

**Fig. 2:**
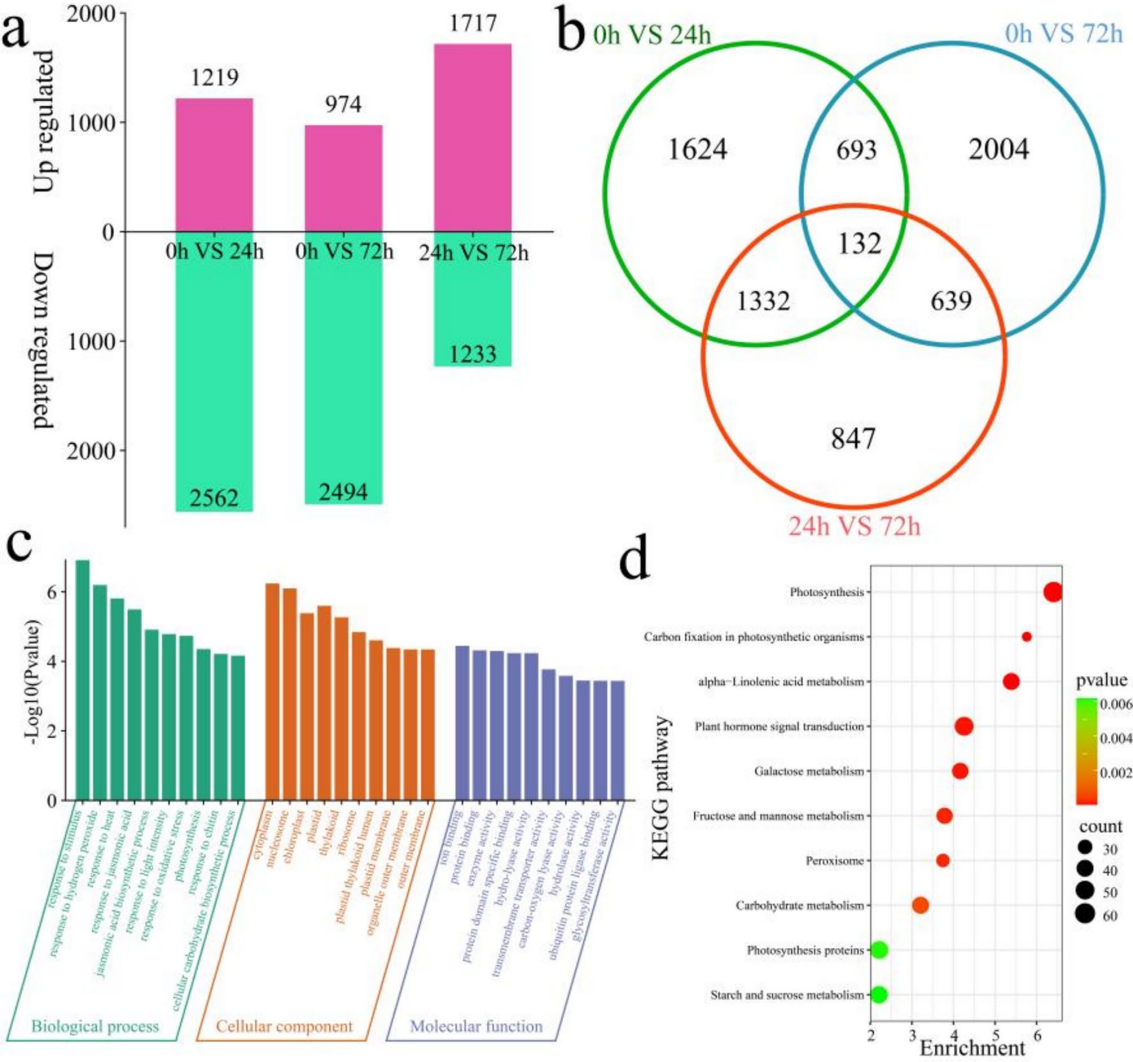
Number and enrichment analysis of DEGs at different time points after 1910 inoculation. (**a**) Number of up- and downregulated DEGs at different time points after 1910 inoculation, (**b**) Venn diagram of specific and common DEGs at different time points after 1910 inoculation, (**c**) GO enrichment analysis of all DEGs at different time points after 1910 inoculation, (**d**) KEGG enrichment analysis of all DEGs at different time points after 1910 inoculation.

To analyze the regulatory mode of Korla fragrant pear against fire blight infection, the DEGs of the disease-resistant line 1910 at different stages of fire blight infection were first identified (Fig. 2a). A total of 3781 DEGs were identified at 0 h vs. 24 h, of which 1219 were upregulated and 2562 were downregulated. A total of 3468 DEGs were identified at 0 h vs. 72 h, of which 974 were upregulated and 2494 were downregulated. A total of 2950 DEGs were identified at 24 h vs. 72 h, of which 1717 were upregulated and 1233 were downregulated. A total of 7271 DEGs were identified at different times after inoculation in 1910, including 132 common DEGs, 1624 of which were differentially expressed only at 0 h vs. 24 h, 2004 of which were differentially expressed only at 0 h vs. 72 h, and 847 of which were differentially expressed only at 24 h vs. 72 h (Fig. 2b). GO and KEGG enrichment analyses were performed on the 7271 DEGs (Fig. 2c and d). GO enrichment analysis revealed that the biological process terms included response to stimulus, response to hydrogen peroxide, response to heat, response to jasmonic acid, jasmonic acid biosynthetic process, response to light intensity, response to oxidative stress, photosynthesis, response to chitin and cellular carbohydrate biosynthetic process (Fig. 2c). The molecular function terms included ion binding, protein binding, enzyme activity, protein domain-specific binding, hydrolyase activity, transmembrane transporter activity, carbon‒oxygen lyase activity, hydrolase activity, ubiquitin protein ligase binding and glycosyltransferase activity (Fig. 2c). KEGG enrichment revealed enrichment in alpha-linolenic acid metabolism, galactose metabolism, fructose and mannose metabolism, plant hormone signal transduction, peroxisome, photosynthesis, photosynthesis proteins, carbohydrate metabolism, starch and sucrose metabolism and carbon fixation in the photosynthetic organism pathways (Fig. 2d and Table S3).

**Fig. 3:**
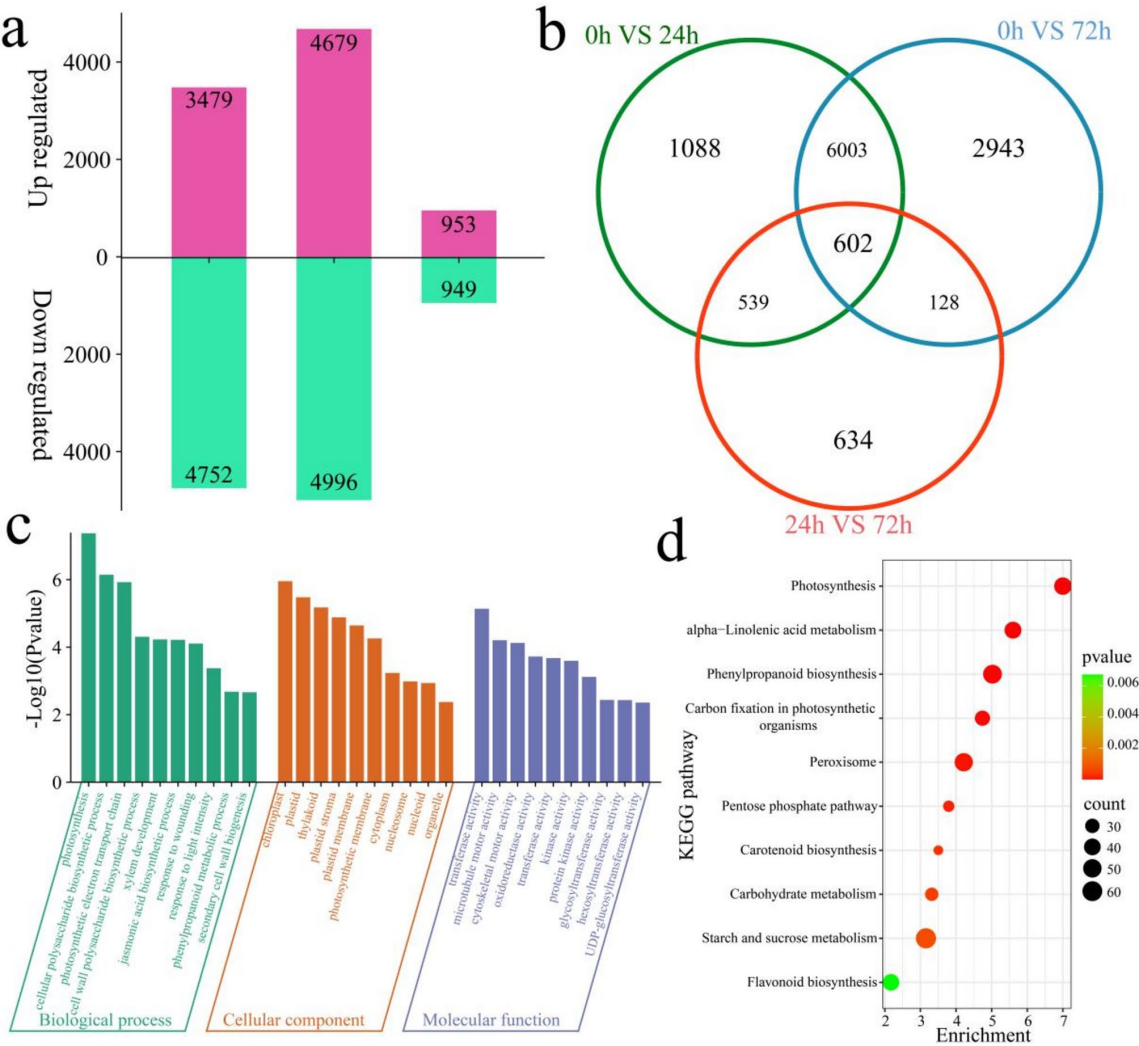
Number and enrichment analysis of DEGs at different time points after KFP inoculation. (**a**) Number of up- and downregulated DEGs at each time point after KFP inoculation, (**b**) Venn diagram of specific and common DEGs at each time point after KFP inoculation, (**c**) GO enrichment analysis of all DEGs at different time points after KFP inoculation, (**d**) KEGG enrichment analysis of all DEGs at different time points after KFP inoculation.

In the susceptible line KFP, 11,937 DEGs were identified at different stages of fire blight inoculation (Fig. 3a). A total of 8231 DEGs were identified at 0 h vs. 24 h, of which 3479 were upregulated and 4752 were downregulated. A total of 9675 DEGs were identified at 0 h vs. 72 h, of which 4679 were upregulated and 4996 were downregulated. A total of 1902 DEGs were identified at 24 h vs. 72 h, of which 953 were upregulated and 949 were downregulated. Among all 11,937 DEGs, 602 were common DEGs, 1088 of which were differentially expressed only at 0 h vs. 24 h, 2943 of which were differentially expressed only at 0 h vs. 72 h, and 634 of which were differentially expressed only at 24 h vs. 72 h (Fig. 3b). GO and KEGG enrichment analyses were performed on 11,937 DEGs (Fig. 3c and d). GO enrichment analysis revealed that the enriched biological processes included photosynthesis, the cellular polysaccharide biosynthetic process, the photosynthetic electron transport chain, the cell wall polysaccharide biosynthetic process, xylem development, the jasmonic acid biosynthetic process, the response to wounding, the response to light intensity, the phenylpropanoid metabolic process and secondary cell wall biogenesis (Fig. 3c). The molecular functions included transferase activity, microtubule motor activity, cytoskeletal motor activity, oxidoreductase activity, transferase activity, kinase activity, protein kinase activity, glycosyltransferase activity, hexosyltransferase activity, and UDP-glucosyltransferase activity (Fig. 3c). The enriched KEGG terms were related mainly to photosynthesis; carbon fixation in photosynthetic organisms; the pentose phosphate pathway; alpha-linolenic acid metabolism; carotenoid biosynthesis; carbohydrate metabolism; and the peroxisome, flavonoid biosynthesis, starch and sucrose metabolism and phenylpropanoid biosynthesis pathways (Fig. 3d).

### Differential expression analysis among lines

**Fig. 4:**
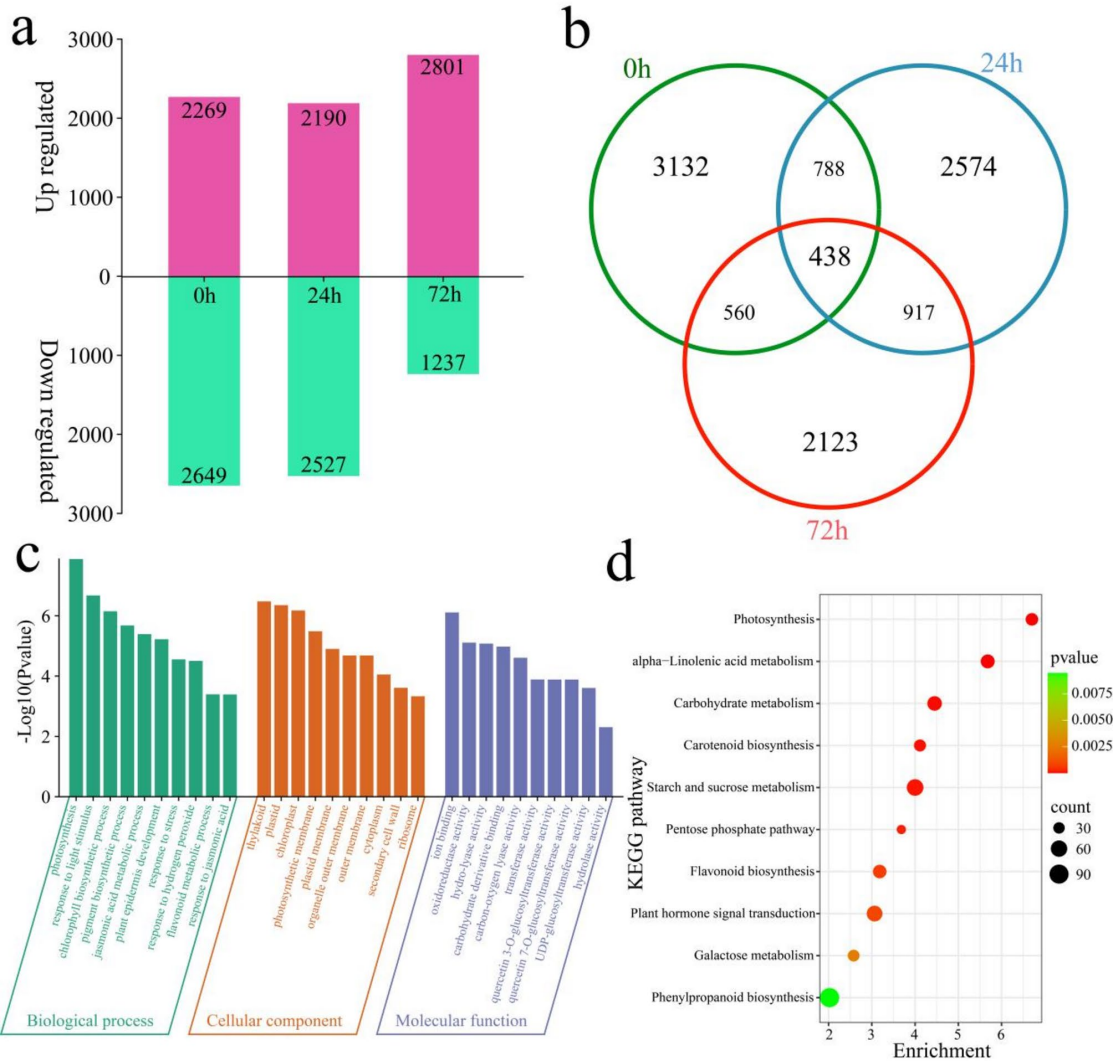
Number and enrichment analysis of DEGs at the same time points after inoculation with KFP and 1910. (**a**) Number of up- and downregulated DEGs at the same time points after KFP and 1910 inoculation. (**b**) Venn diagram of specific and common DEGs at the same time points of KFP and 1910 inoculation. (**c**) GO enrichment analysis of all DEGs at the same time points of KFP and 1910 inoculation. (**d**) KEGG enrichment analysis of all DEGs at the same time points of KFP and 1910 inoculation.

A total of 10,532 DEGs were identified in the susceptible accession KFP and the resistant accession 1910 at the time of inoculation with fire blight (Fig. 4a). A total of 4918 DEGs were identified at 0 h, of which 2269 were upregulated and 2649 were downregulated. A total of 4717 DEGs were identified within 24 h, of which 2190 were upregulated and 2527 were downregulated. A total of 4038 DEGs were identified within 72 h, of which 2801 were upregulated and 1237 were downregulated. Among all 10,532 DEGs, 438 were common DEGs, 3132 of which were differentially expressed at only 0 h, 2574 at only 24 h, and 2123 at only 72 h (Fig. 4b). GO and KEGG enrichment analyses were performed on 11,937 DEGs (Fig. 4c and d). The biological processes of GO enrichment analysis included photosynthesis, response to light stimulus, chlorophyll biosynthetic process, pigment biosynthetic process, jasmonic acid metabolic process, plant epidermis development, response to stress, response to hydrogen peroxide, flavonoid metabolic process and response to jasmonic acid (Fig. 4c). The molecular functions included ion binding, oxidoreductase activity, hydrolyase activity, carbohydrate derivative binding, carbon‒oxygen lyase activity, transferase activity, quercetin 3-O-glucosyltransferase activity, quercetin 7-O-glucosyltransferase activity, UDP-glucosyltransferase activity and hydrolase activity (Fig. 4c). The enriched KEGG terms were related mainly to photosynthesis, carotenoid biosynthesis, plant hormone signal transduction, the pentose phosphate pathway, alpha-linolenic acid metabolism, galactose metabolism, carbohydrate metabolism, flavonoid biosynthesis, phenylpropanoid biosynthesis and starch and sucrose metabolism pathways (Fig. 4d).

### Cluster analysis

**Fig. 5:**
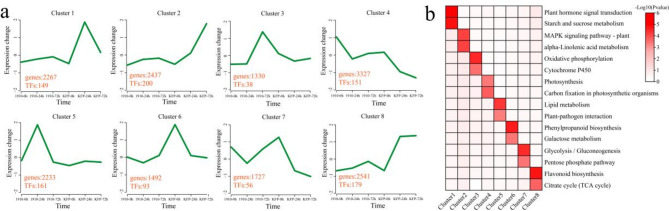
Number and enrichment analysis of DEGs at the same time points of KFP and 1910 inoculation. (**a**) Line graph of cluster analysis of DEGs; the numbers represent the number of DEGs and TFs in each cluster. (**b**) Heatmap of the results of the KEGG enrichment analysis of each cluster.

A total of 8 clusters were identified for all (17,354) DEGs via k-means, and KEGG pathway annotations were performed for each individual cluster (Fig. 5a and b). Cluster 1 included 2267 DEGs, including 149 transcription factors (TFs), whose expression level was highest at 24 h after KFP inoculation, remained essentially unchanged in 1910, and was significantly enriched in the plant hormone signal transduction and starch and sucrose metabolism pathways. Cluster 2 included 2437 DEGs, including 200 TFs whose expression levels increased after KFP inoculation and reached a maximum at 72 h, and their expression remained basically unchanged in 1910. These genes were significantly annotated in the MAPK signaling pathway and alpha-linolenic acid metabolism pathways. Cluster 3 included 1330 DEGs, including 38 TFs, with the highest expression level at 72 h in 1910 bacteria, and the expression in KFP remained basically unchanged and was significantly annotated in the oxidative phosphorylation and cytochrome P450 pathways. Cluster 4 included 3327 DEGs, including 151 TFs whose expression levels decreased after the inoculation of KFP and 1910, reached the minimum value at 72 h, and were significantly annotated in the photosynthesis and carbon fixation in photosynthetic organisms pathway. Cluster 5 included 2233 DEGs, including 161 TFs, with the highest expression level at 24 h after 1910 inoculation, and the expression in KFP remained basically unchanged and was significantly annotated in the lipid metabolism and plant‒pathogen interaction pathways. Cluster 6 included 1492 DEGs, including 93 TFs, and presented the highest expression level at 0 h after KFP inoculation. The expression level gradually decreased with inoculation, remained essentially unchanged in 1910, and was significantly associated with the phenylpropanoid biosynthesis and galactose metabolism pathways. Cluster 7 included 1727 DEGs, including 56 TFs. The expression level was highest at 0 h after KFP inoculation and gradually decreased with inoculation. After the expression level decreased at 24 h in 1910, the expression level increased at 72 h and was significantly associated with the glycolysis/gluconeogenesis and pentose phosphate pathways. Cluster 8 included 2541 DEGs, including 179 TFs, with the highest expression levels at 24 h and 72 h after KFP inoculation, and the expression in 1910 remained basically unchanged and was significantly annotated in the flavonoid biosynthesis and citrate cycle (TCA cycle) pathway.

### TF analysis

**Fig. 6:**
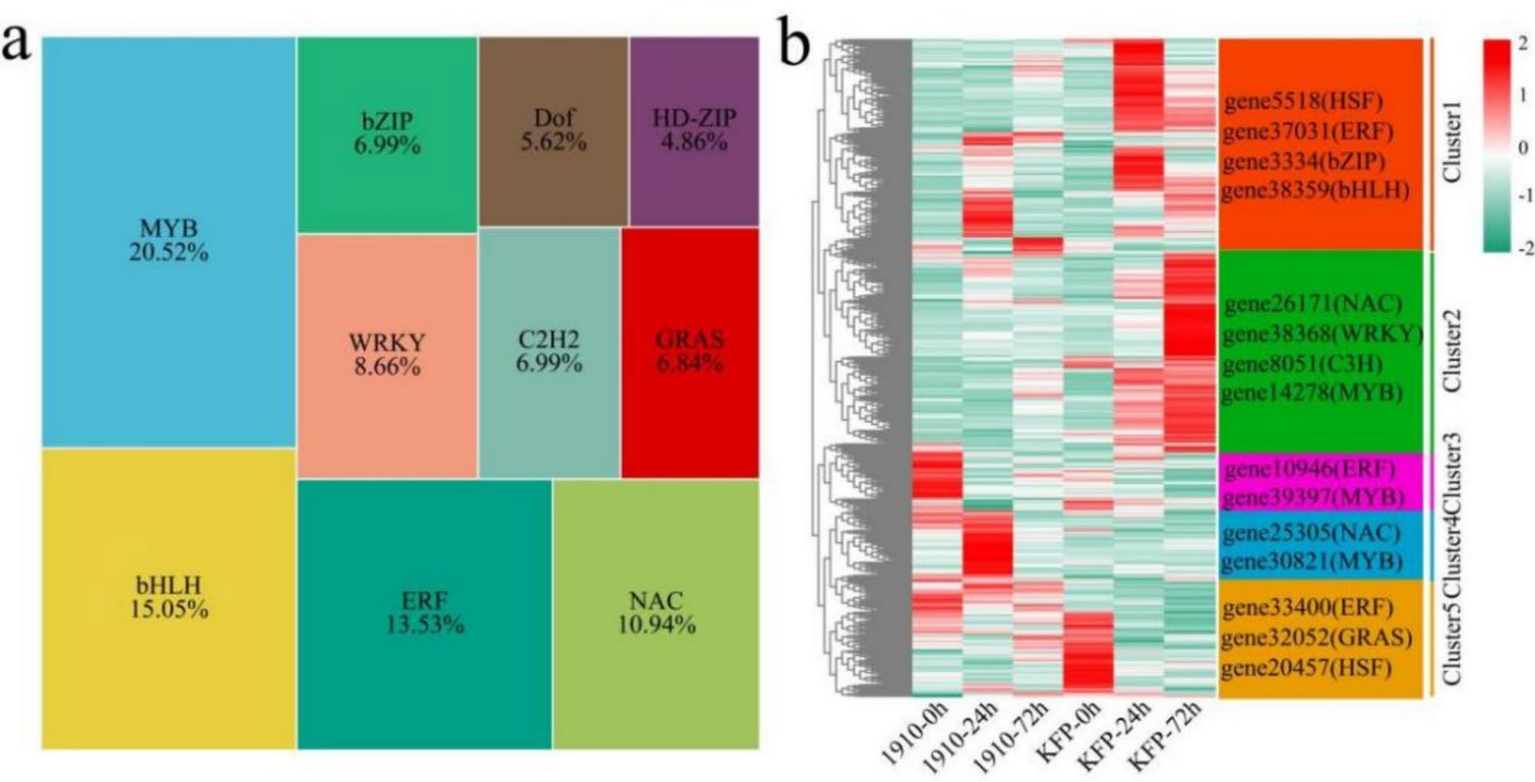
Proportion of differentially expressed TFs and cluster analysis of expression patterns. (**a**) Percentage of differentially expressed TFs. (**b**) Cluster analysis of differentially expressed TFs.

A total of 1027 differentially expressed transcription factors were identified among the 17,354 DEGs, and the top 10 TFs were bHLH, MYB, ERF, NAC, WRKY, bZIP, C2H2, GRAS, Dof and HD-ZIP (Fig. 6a). MYB transcription factors can protect against the infection of pathogenic microorganisms such as fungi, bacteria, and viruses by regulating the expression of defense genes downstream of plant defense responses or by participating in the synthesis of secondary metabolites^22^. AP2/ERF is involved in plant disease resistance by acting downstream of the mitogen-activated protein kinase (MAPK) cascade to regulate the expression of genes associated with hormone signaling pathways, the biosynthesis of secondary metabolites, and the formation of physical barriers in the MAPK pathway. The signal transduction pathway of the plant disease resistance response is established by the disease resistance regulatory gene NPR1 and the disease resistance-related gene PR^23^. WRKY transcription factors can regulate or initiate gene expression by binding to W-box elements in the NPR1 and PR promoters, which in turn activate plant defense responses^24^. The absolute values ​​of the log_2_fold changes in three WRKY transcription factors *(gene6486*,* gene23487* and *gene38368*) were greater than 5. A total of 5 statistically significant clusters were identified among the 1027 differentially expressed TFs via hierarchical clustering (Fig. 6b). The expression level of Cluster 1 increased after inoculation with KFP and 1910, and four TOP TFs were identified, namely, gene5518 (HSF), gene37031 (ERF), gene3334 (bZIP) and gene38359 (bHLH). The expression level of Cluster 2 increased 72 h after KFP inoculation and reached the maximum value. The expression level in 1910 remained essentially unchanged. Four TOP TFs were identified, namely, gene26171 (NAC), gene38368 (WRKY), gene8051 (C3H) and gene14278 (MYB). The expression level of Cluster 3 decreased after inoculation at 1910, and the expression level in KFP remained essentially unchanged. Two TOP TFs were identified: gene10946 (ERF) and gene39397 (MYB). The expression level of Cluster 4 was the highest at 24 h after inoculation in 1910, and the expression level in KFP remained basically unchanged. Two TOP TFs were identified: gene25305 (NAC) and gene30821 (MYB). The expression level of Cluster 5 decreased after KFP inoculation but remained essentially unchanged in 1910. Three TOP TFs were identified: gene33400 (ERF), gene32052 (GRAS) and gene20457 (HSF).

### WGCNA

**Fig. 7:**
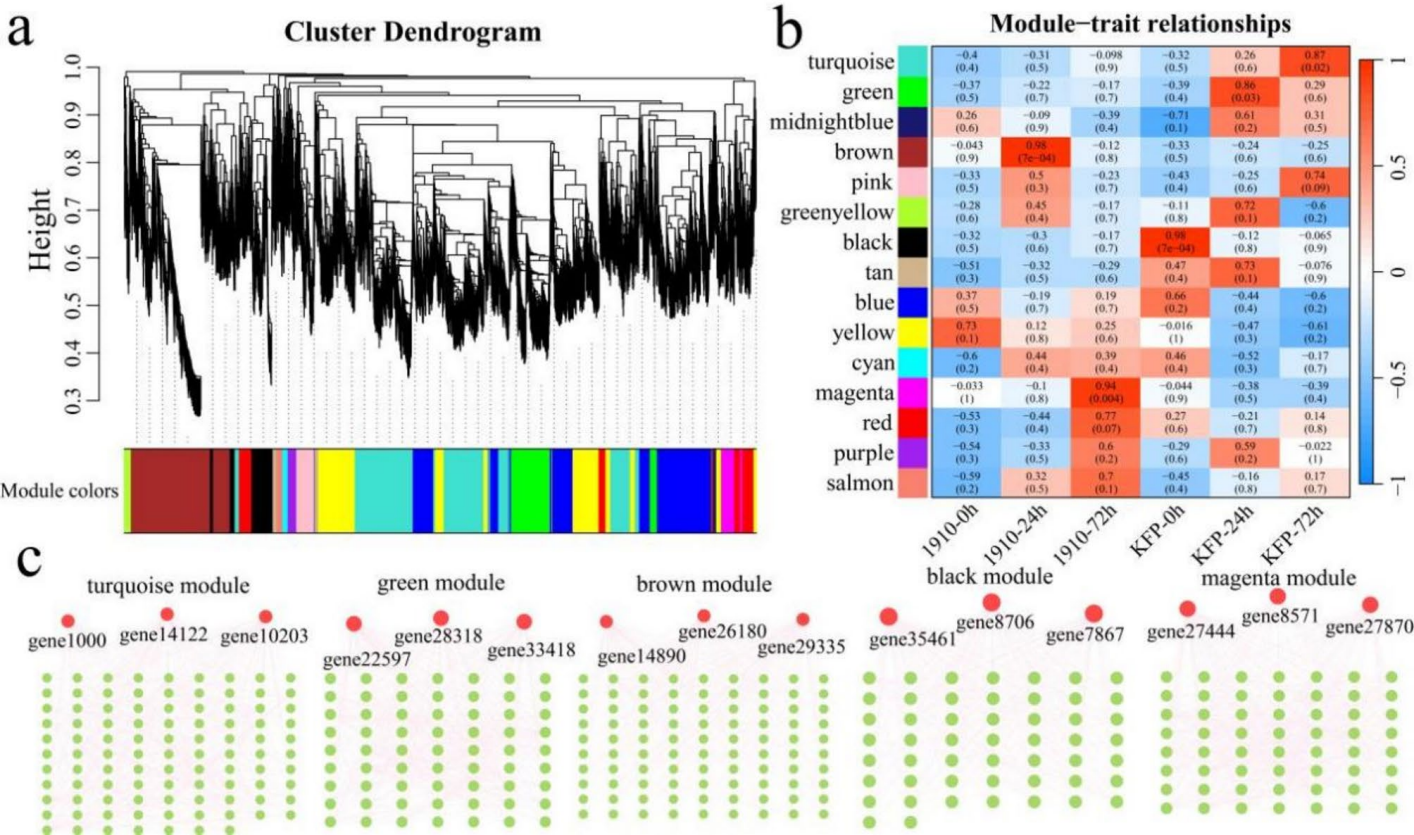
WGCNA and selection of candidate genes. (**a**) WGCNA module hierarchical clustering tree diagram; different modules are represented by different colors; (**b**) correlation and significance heatmaps between samples and modules; (**c**) module gene interaction network diagram.

To construct a gene coexpression network of Korla fragrant pear resistance to fire blight and mine candidate genes, an expression matrix of 17,352 DEGs was used for WGCNA to construct a gene coexpression network of Korla fragrant pear resistance to fire blight. A total of 15 different coexpression modules were identified. The largest and smallest modules were the turquoise and dark red modules, containing 3355 and 49 genes, respectively (Fig. 7a). According to the correlation results between the modules and different periods of fire blight inoculation, the turquoise module was significantly highly correlated with KFP at 72 h, the green module was significantly highly correlated with KFP at 24 h, the brown module was significantly highly correlated with KFP at 19–10 24 h, the black module was significantly highly correlated with KFP at 0 h, and the magenta module was significantly highly correlated with KFP at 19–10 72 h (Fig. 7b). By calculating the kME (eigengene connectivity) value of each module gene, the gene with the highest absolute kME value was used as the hub gene of each module. In each module, the three genes with the highest degree of linkage were identified as candidate genes, and 15 candidate genes were ultimately obtained (Fig. 7c). To further explain the relationships between the nine candidate genes and the resistance of Korla fragrant pear to fire blight, the functions of the candidate genes were annotated to homologous *Arabidopsis* genes (Table 1). Among them, five genes encode transcription factors, namely, gene1000 (C3H), gene26180 (MYB), gene29335 (bHLH), gene33418 (TCP) and gene7867 (MYB). *Gene10203* encodes fructose-1-6-bisphosphatase, which is involved mainly in carbon fixation in photosynthetic organisms; *gene14122* encodes chlacone isomerase, which is involved mainly in flavonoid biosynthesis; *and genes14890 and 8706* encode lipoxygenase and allene oxide cyclase, respectively, which are involved mainly in jasmonic acid biosynthesis. The *genes 22,597* and *27,444* encode the photosystem II CP47 chlorophyll apoprotein and cytochrome b6-f complex iron‒sulfur subunit, respectively, which are involved mainly in photosynthesis. *Gene27870* encodes L-type lectin domain-containing receptor kinase IV.1, which is involved mainly in starch and sucrose metabolism. The *gene28318* encodes leucine-rich repeat receptor-like kinases, which are involved mainly in the MAPK signaling pathway. *Gene8571* and *gene35461* encode leucine-rich repeat receptor-like proteins that are involved mainly in plant‒pathogen interactions.

**Table 1:** Annotation information of candidate genes.

Gene id	Gene name	*A. thaliana* homologous genes	Functional annotation
gene1000	C3H	AT1G68200	–
gene10203	FBP	AT3G54050	Carbon fixation in photosynthetic organisms
gene14122	CHI	AT5G05270	Flavonoid biosynthesis
gene14890	LOX	AT1G67560	Jasmonic acid biosynthesis
gene22597	psbB	AT1G63770	Photosynthesis
gene26180	MYB	AT4G39250	Plant‒pathogen interaction
gene27444	petC	AT1G26400	Photosynthesis
gene27870	LECRK	AT5G13290	Starch and sucrose metabolism
gene28318	LRR-RLK	AT5G48740	MAPK signaling pathway
gene29335	bHLH	AT5G42390	–
gene33418	TCP	AT5G51910	Circadian rhythm
gene35461	LRR-RLP	AT4G19045	Plant‒pathogen interaction
gene7867	MYB	AT1G73410	–
gene8571	RLP	AT4G19045	Plant‒pathogen interaction
gene8706	AOC	AT3G25760	Jasmonic acid biosynthesis

### qRT‒PCR

**Fig. 8:**
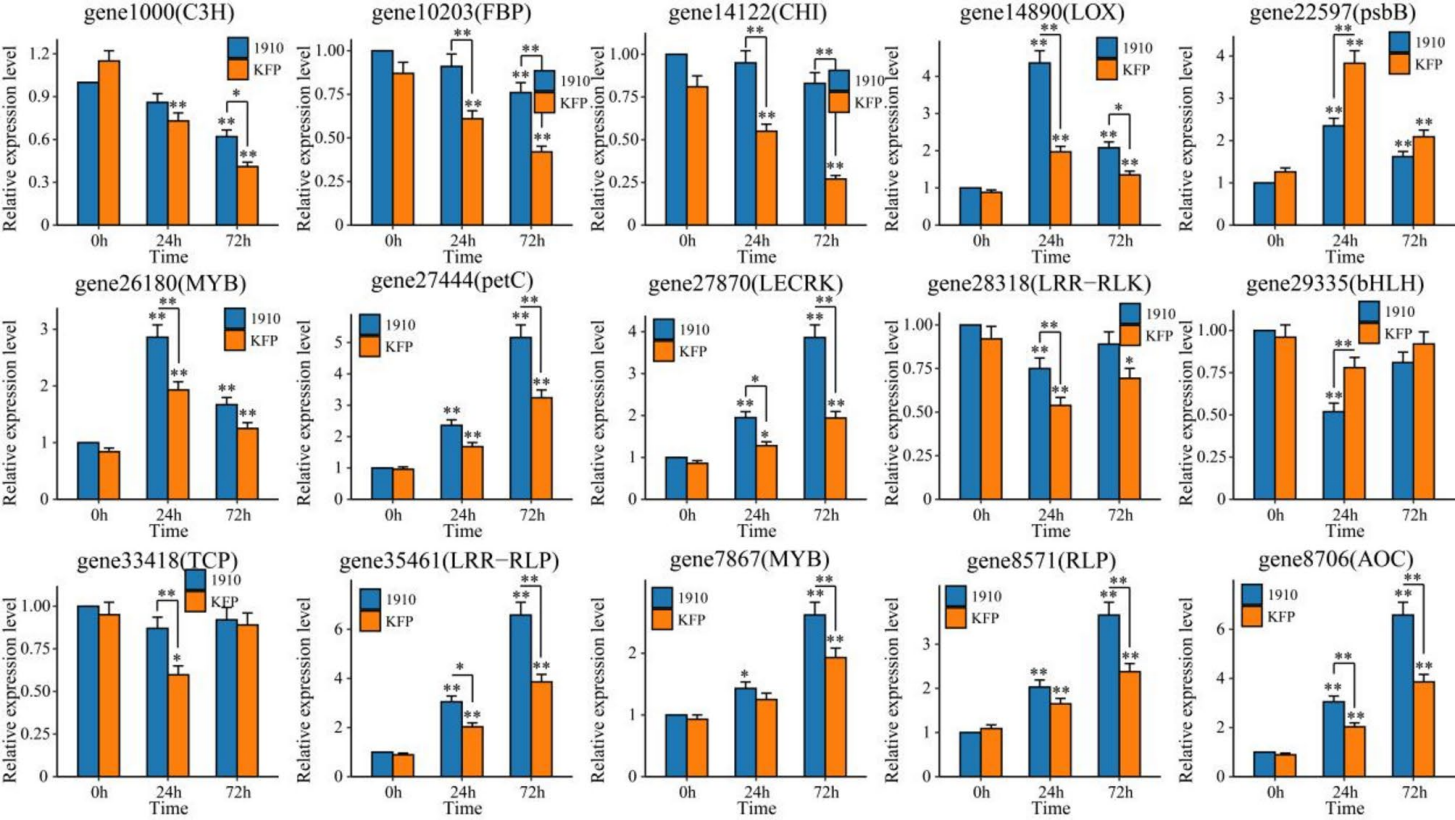
Expression patterns of the hub genes at different time points after inoculation with fire blight. The error bars represent the means of three replicates ± SDs (*P < 0.05; **P < 0.01).

qRT‒PCR was used to further detect the expression patterns of these 15 Hub genes under fire blight infection conditions (Fig. 8 and Table S4). Compared with those at 0 h, the expression levels of 6 genes (*gene1000*, *gene10203*, *gene14122*, *gene28318*, *gene29335* and *gene33418*) decreased significantly after inoculation with fire blight. Except for *gene29335*, the expression levels of the other 5 genes in KFP were significantly lower than those in 1910. The expression levels of 9 genes (*gene14890*, *gene22597*, *gene26180*, *gene27444*, *gene27870*, *gene35461*, *gene7867*, *gene8571* and *gene8706*) increased significantly after inoculation with fire blight. Except for *gene22597*, the expression levels of the other 8 genes in 1910 were significantly greater than those in KFP. The fold changes in *genes 14,890*,* 27,444*,* 35,461 and 8706* after inoculation with fire blight were greater than 4, which indicated that these four genes may play important roles in the resistance of Korla fragrant pear to fire blight.

## Discussion

### Effects of fire blight on Korla Pear and the important role of ROS in its disease resistance

Owing to the complexity and diversity of plant resistance mechanisms, plants have different response rates to foreign pathogens^25^. As the genetic background of Pyrus is complex and variable, clarifying the regulatory mechanism by which Pyrus protects against the invasion of fire blight pathogens into trees is important for the prevention and control of Pyrus fire blight. The Korla fragrant pear tree is highly susceptible. During the growth period of a pear tree, the inflorescence, young fruits and branches are most susceptible to disease^4^. After the pathogen infects plant tissue, it exudes fungal pus^26^. Many pathogens in fungal pus can be spread through insects, pollination, wind and rain, pruning and other means^27^. The plant-induced resistance mechanism involves a combination of factors, including antioxidant enzymes, lignin-related phenylpropanoid enzymes, the expression of disease resistance-related amino acids and proteins, the synthesis of phytoalexins, the secretion of endogenous hormones, etc^4,28^. During the *Botryosphaeria dothidea* infection stage, pear trees positively regulate resistance to *B. dothidea* through chitinase activity and attenuation of ROS accumulation^29^. However, at present, few reports exist on the core pathways and genes involved in the resistance mechanism triggered by Korla fragrant pear resistance to fire blight. It is highly important to conduct in-depth research on the regulatory mechanism of the response of Korla fragrant pear to fire blight. To this end, this study conducted RNA-seq sequencing of the susceptible line KFP and the resistant line 1910 at different time points after inoculation with fire blight. More DEGs were identified in the susceptible material KFP, indicating that these DEGs may be more conducive to the invasion and reproduction of fire blight or that the immune response it produces is ineffective. DEGs associated with the biological process of response to hydrogen peroxide were significantly annotated. The production of ROS can synergistically drive plant disease resistance and allergic death. In addition, ROS are involved in the lignification of cell walls and the cross-linking of proteins and cell walls, thereby strengthening plant cell walls to resist the invasion of pathogens. These findings suggest that after being invaded by fire blight, Korla pear may be able to trigger the production of the ROS intermediate hydrogen peroxide (H_2_O_2_), drive the cross-linking of cell walls, and induce the production of downstream substances involved in cell protection and defense.

### The jasmonic acid signaling pathway plays an important role in the response of Korla Pear to fire blight

JA and its derivatives are lipid-derived molecules. When plants are attacked by pathogens or insects, they quickly synthesize JA and its derivatives through the oxylipin pathway^30^. JA synthesis begins with the release of α-linolenic acid from membrane lipids, which is synthesized by various enzymes in the lipoxygenase pathway^31^. Currently, two lipases, DAD1 (DEFECTIVE IN ANTHER DEHISCENCE) and DGI (DONGLE), are reportedly involved in the release of α-linolenic acid during the synthesis of JA^32^. The jasmonic acid synthesis-deficient mutants *fad3/fad7/fad8dadl*, *dde1/opr3* and *dde2/aos* in *Arabidopsis thaliana* have significantly weakened resistance to pathogenic fungi^33–35^. JA can also be transported over long distances through vascular bundles, transmitting pathogen invasion signals from the infection site to the entire plant and causing systemic resistance in the entire plant^36^. JA can also induce the synthesis of large amounts of substances such as lignin, flavonoids and terpenes in plants after pathogen invasion to improve their disease resistance^37^. When the JA content in the plant body increases, it can also accelerate the growth rate of the plant’s epidermal hairs and thicken the secondary wall, making it difficult for pathogens to invade and colonize^38^. The two rate-limiting enzymes, AOS and AOC, play decisive roles in JA synthesis^39^. The gray mold lesions of the jasmonic acid-insensitive mutant (*jai1*) in tomato are three times larger than those of the wild type^40^. *SlMYC2* positively regulates plant resistance to Botrytis cinerea by regulating pathogen response genes^41^. Conversely, *SlMYC2*-RNAi plants were significantly more sensitive to Botrytis cinerea than were WT plants. In *Arabidopsis thaliana*, *AtNAC019* and *AtNAC055* participate in the JA-mediated defense response by transcriptionally regulating the expression of the JA-induced defense genes *VSP1* and *LOX2*^42^. Through differential expression analysis, we found that DEGs were significantly enriched in alpha-linolenic acid metabolism and jasmonic acid metabolic processes, and two JA biosynthesis genes (*gene14890* and *gene8706*) were screened by WGCNA as candidate genes for Korla fragrant pear resistance to fire blight. The expression levels of the *genes 14,890* and *8706* in the resistant materials were significantly greater than those in the susceptible materials, and the expression levels significantly increased after inoculation. The fold changes of *genes 14,890* and *8706* before and after inoculation were greater than those of other genes in the JA biosynthesis pathway, indicating that *genes 14,890* and *8706* play important roles in improving the resistance of Korla fragrant pear to fire blight via the JA pathway.

### Flavonoid pathway plays an important role in the response of Korla fragrant Pear to fire blight

**Fig. 9:**
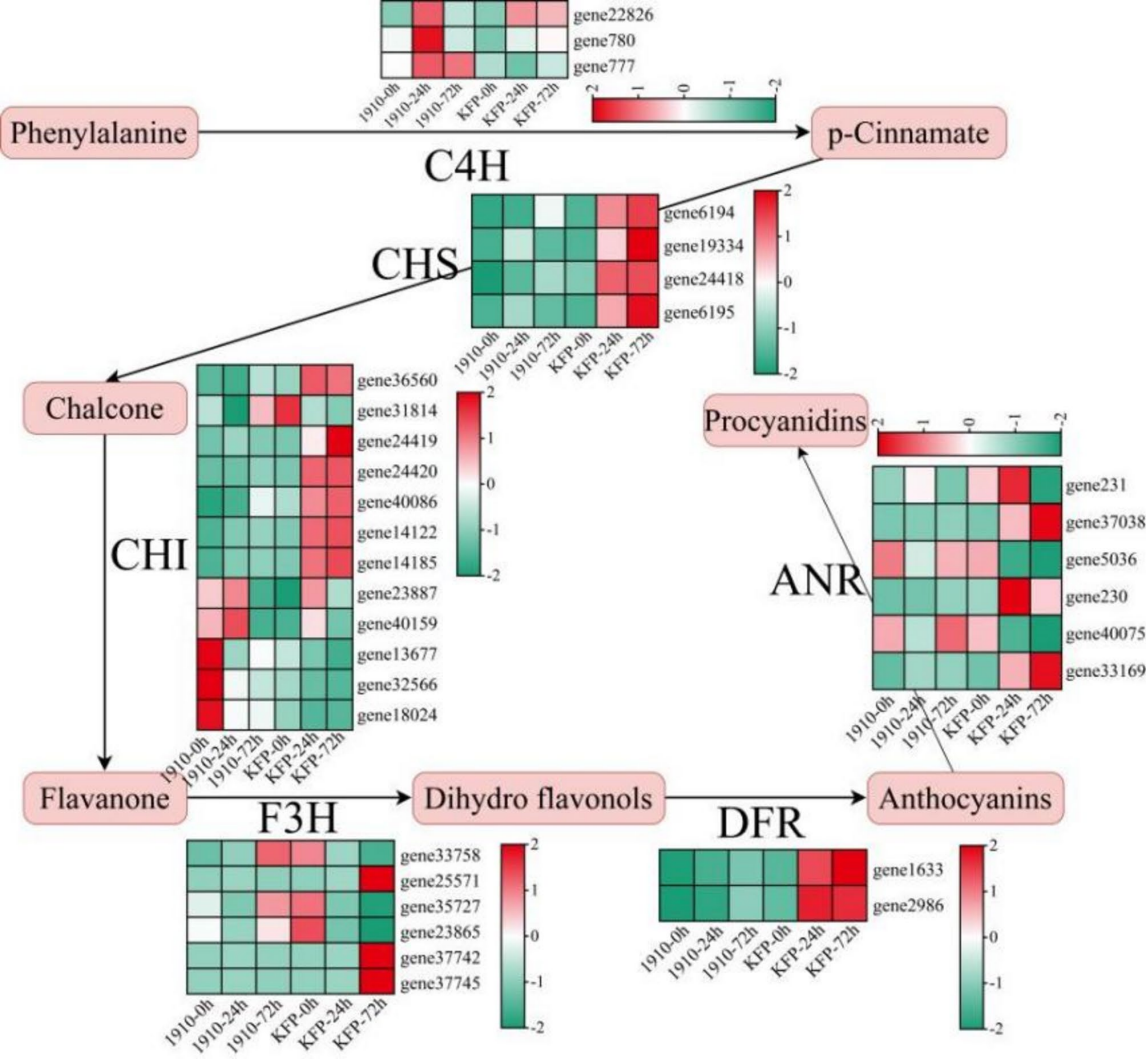
Expression patterns of flavonoid pathway genes differentially expressed during the development of fire blight disease in Korla fragrant pear.

Flavonoid synthesis is carried out through the phenylpropanoid pathway, which is synthesized through the joint action of related genes such as PAL, CHI and CHS^43^. In plants, flavonoid metabolites can have antioxidant effects, such as resistance to diseases and insects, resistance to ultraviolet rays, and free radical scavenging. Therefore, flavonoid metabolites are mostly distributed in the epidermal cell layer of plant leaves and in pollen and apical meristems^44^. Moreover, when plants are attacked by pathogens or pests, anthocyanins are produced in the body, which mainly accumulate in the fruits, endocarps, and glandular hairs of the plants, thereby protecting the plants from harm caused by microorganisms and animals. Antioxidation is one of the most prominent effects of flavonoids^45^. It can neutralize free radicals, hydrogen peroxide and other oxidizing substances; protect plant cells from oxidative damage; and thus improve plant disease resistance. It can also inhibit the growth and reproduction of pathogens, mainly by destroying the cell wall or cell membrane of pathogenic microorganisms, interfering with their metabolism and growth, thereby inhibiting the infection and spread of pathogens and protecting plants from diseases^46^. It can also stimulate plants to produce a series of disease-resistant substances, such as antibacterial proteins and antioxidant enzymes, to increase plant resistance to pathogenic microorganisms^47^. Moreover, flavonoids can also regulate plant immune response transduction pathways, activate plant defense mechanisms, and quickly respond to pathogen infection, thereby improving plant disease resistance. For example, as the content of anthocyanins in cotton increases, its resistance to wilt disease becomes increasingly strong^48^. Through enrichment analysis of the DEGs, we found that the flavonoid biosynthesis and phenylpropanoid biosynthesis pathways were significantly enriched (Fig. 9). Moreover, WGCNA revealed a gene (*gene14122* (CHI)) that regulates flavonoid biosynthesis. The expression level of *gene 14,122* in disease-resistant materials was significantly greater than that in susceptible materials, and the expression level decreased significantly after inoculation. We also screened 2 MYB (*gene7867* and *gene26180*) and 1 bHLH (*gene29335*) TF, and MBW (MYB-bHLH-WDR) complexes, such as *MYB123*, *MYB5*,* bHLH001*,* bHLH002* and *bHLH042*^49^, could also regulate the content of plant flavonoids. The expression levels of *the genes7867* and *26,180* in the resistant materials were significantly greater than those in the susceptible materials, and the expression levels were significantly greater after vaccination. These findings indicate that the flavonoid pathway plays an important role in the resistance of Korla fragrant pear to fire blight. We screened the *gene 14,122* mentioned above, which may play an important role in the resistance of Korla fragrant pear to fire blight. In the later stage of Korla pear infection, metabolome sequencing is still needed to explore the relationships between these genes and changes in metabolite content to provide information for improving the resistance of Korla pear to fire blight through the flavonoid pathway.

### Photosynthesis and sugar metabolism play important roles in the response of Korla Pear to fire blight

Photosynthesis is a very important physiological process in plants that converts carbon dioxide and water into organic matter and oxygen through light energy^50^. In this process, plants produce large amounts of energy and nutrients to maintain the energy metabolism required for growth and survival. In addition, photosynthesis plays a vital role in plant disease resistance^51^. First, the organic matter produced by photosynthesis can provide nutrients to plants, thereby increasing their ability to grow and develop. Strong plants are more likely to resist attack by pathogens because they have more developed defense response systems and resistance mechanisms^52^. In addition, the oxygen produced by photosynthesis also helps plants fight pathogens because oxygen can improve the redox state within plant cells and enhance the effectiveness of the immune response. Second, the energy produced by photosynthesis can help plants accelerate the repair of damaged tissues^53^. When plants are attacked by pathogens, they immediately activate their own defense mechanisms, such as increasing the rate of disease resistance-related substance production, to resist the invasion of pathogens. With the energy generated by photosynthesis, plants can repair damaged tissue more quickly and return to a healthy state. The organic matter and oxygen produced through photosynthesis can change the chemical composition and redox state of the surrounding environment, thereby affecting the growth and reproduction of pathogens^54^. The organic matter produced by photosynthesis, such as cellulose and lignin, can also be used to increase the strength of the cell wall^55^. Strengthening plant cell walls can increase the mechanical strength and stress resistance of plant tissues, increasing the resistance of plants to invasion by external pathogens and thereby improving plant disease resistance^56^. Our analysis revealed the importance of photosynthesis, starch and sucrose metabolism and carbon fixation pathways in the resistance of Korla fragrant pear to fire blight, and 4 of the 15 candidate genes screened (*gene10203* (FBP), *gene22597* (psbB), *gene27444* (petC) and *gene27870* (LECRK)) were located in these pathways. The expression level of *gene10203* in disease-resistant materials was significantly greater than that in susceptible materials, and the expression level decreased significantly after inoculation. The expression levels of *genes 27,444* and *27,870* in the resistant materials were significantly greater than those in the susceptible materials, and the expression levels were significantly greater after inoculation. In summary, this study analyzed RNA-seq data at different time points after Korla fragrant pear inoculation with fire blight, emphasized the important role of flavonoid biosynthesis, photosynthesis, and hormone pathways in Korla resistance to fire blight, and screened 15 candidate genes that can be used as the focus of subsequent research. Notably, this study also has several potential limitations in that the resistance mechanism was inferred through only transcriptome data. In the future, metabolome protein and other omics data are needed to obtain a more comprehensive understanding of the molecular mechanism of Korla pear response to fire blight.

## Conclusion

In this study, RNA-seq was performed on Korla fragrant pear at three time points after inoculation with fire blight. A total of 17,354 DEGs containing 1,027 TFs were identified between and within lines and clustered into eight clusters via the k-means clustering algorithm, and the pathways of each cluster were annotated. The importance of flavonoid biosynthesis, photosynthesis and plant hormone pathways in Korla resistance to fire blight was emphasized, which is highly important for future improvement strategies for the resistance of Korla fragrant pear to fire blight. In addition, 15 candidate genes were screened, but further functional verification is needed to determine their exact role in Korla fragrant pear resistance to fire blight. These research results provide a theoretical basis for a deeper understanding of the molecular mechanism of Korla fragrant pear resistance to fire blight and provide new genetic resources for the study of Korla fragrant pear resistance to fire blight.

## Electronic supplementary material

Below is the link to the electronic supplementary material.

Supplementary Material 1

Supplementary Material 2

Supplementary Material 3

Supplementary Material 4

## Data Availability

The RNA-seq data presented in the study are deposited in the NCBI repository under accession number PRJNA1204458.
